# Zonation of the active methane-cycling community in deep subsurface sediments of the Peru trench

**DOI:** 10.3389/fmicb.2023.1192029

**Published:** 2023-05-12

**Authors:** Mark A. Lever, Marc J. Alperin, Kai-Uwe Hinrichs, Andreas Teske

**Affiliations:** ^1^Department of Marine Science, Marine Science Institute, University of Texas at Austin, Port Aransas, TX, United States; ^2^Earth, Marine and Environmental Sciences, University of North Carolina at Chapel Hill, Chapel Hill, NC, United States; ^3^Organic Geochemistry Group, MARUM-Center for Marine Environmental Sciences and Department of Geosciences, University of Bremen, Bremen, Germany

**Keywords:** deep biosphere, methanogenesis, anaerobic oxidation of methane, subseafloor sediment, ocean drilling, methane hydrate, carbon isotopes, *mcrA*

## Abstract

The production and anaerobic oxidation of methane (AOM) by microorganisms is widespread in organic-rich deep subseafloor sediments. Yet, the organisms that carry out these processes remain largely unknown. Here we identify members of the methane-cycling microbial community in deep subsurface, hydrate-containing sediments of the Peru Trench by targeting functional genes of the alpha subunit of methyl coenzyme M reductase (*mcrA*). The *mcrA* profile reveals a distinct community zonation that partially matches the zonation of methane oxidizing and –producing activity inferred from sulfate and methane concentrations and carbon-isotopic compositions of methane and dissolved inorganic carbon (DIC). *Mcr*A appears absent from sulfate-rich sediments that are devoid of methane, but *mcr*A sequences belonging to putatively methane-oxidizing ANME-1a-b occur from the zone of methane oxidation to several meters into the methanogenesis zone. A sister group of ANME-1a-b, referred to as ANME-1d, and members of putatively aceticlastic *Methanothrix* (formerly *Methanosaeta*) occur throughout the remaining methanogenesis zone. Analyses of 16S rRNA and *mcr*A-mRNA indicate that the methane-cycling community is alive throughout (rRNA to 230 mbsf) and active in at least parts of the sediment column (mRNA at 44 mbsf). Carbon-isotopic depletions of methane relative to DIC (−80 to −86‰) suggest mostly methane production by CO_2_ reduction and thus seem at odds with the widespread detection of ANME-1 and *Methanothrix*. We explain this apparent contradiction based on recent insights into the metabolisms of both ANME-1 and *Methanothricaceae*, which indicate the potential for methanogenetic growth by CO_2_ reduction in both groups.

## Introduction

The detection of active microbial populations to 80 mbsf in Peru Margin sediments during Ocean Drilling Program (ODP) Leg 112 in 1988, was the first demonstration of an deep subseafloor biosphere (Cragg et al., 1990). Since then, numerous studies and multiple lines of evidence from a range of locations have shown a vast microbial biomass in deep subseafloor sediments (for syntheses, see D’Hondt et al., 2004; Kallmeyer et al., 2012; Parkes et al., 2014) with metabolically active cells to at least 1,500 mbsf (Roussel et al., 2008; Inagaki et al., 2015; Heuer et al., 2020), and the existence of a subsurface microbiome that is distinct from that found in marine surface sediments (e.g., Deng et al., 2020; Hoshino et al., 2020).

Several sites sampled during ODP Leg 112 were revisited in 2002 during ODP Leg 201, now 22 years ago, during the first ocean drilling expedition to focus on subseafloor life (D’Hondt et al., 2003). Porewater concentration gradients of microbially consumed electron acceptors such as nitrate or sulfate indicated active microbial populations to depths of >400 mbsf in the sediment column (D’Hondt et al., 2004). Molecular biological studies, e.g., polymerase-chain-reaction (PCR) assays of 16S rRNA genes (Parkes et al., 2005; Inagaki et al., 2006; Webster et al., 2006) and 16S rRNA gene transcripts (Biddle et al., 2006; Sørensen and Teske, 2006), fluorescence-*in-situ*-hybridization (FISH; Mauclaire et al., 2005, Schippers et al., 2005), and metagenomic signatures of whole-genome amplified DNA (Biddle et al., 2008) provided insights into the community structure and metabolic potential of microbial populations. Yet, specific links between microbial activity based on geochemical gradients and microbial identity based on genetic and genomic assays could not be established. For instance, sulfate and methane profiles suggested that sulfate reduction, anaerobic oxidation of methane (AOM), and methanogenesis were all important microbially-driven *in situ* processes (D’Hondt et al., 2004). However, sulfate-reducing, methanogenic, or methane-oxidizing microorganisms were surprisingly rare or absent from clone libraries of transcribed, PCR-amplified 16S rRNA (Biddle et al., 2006; Sørensen and Teske, 2006) and PCR-amplified 16S rRNA genes (Parkes et al., 2005; Inagaki et al., 2006).

Functional genes that encode for enzymes that are unique to certain metabolisms can be targeted to identify microorganisms that are involved in these metabolisms. Functional genes that have been investigated in targeted studies at ODP Leg 201 sites include the gene for dissimilatory sulfite reductase (*dsrAB*), a key enzyme of dissimilatory sulfate reduction (Wagner et al., 2005), the gene for reductive dehalogenase (*rdh*A) of reductive dehalorespiration (Futagami et al., 2009), the gene for formyl tetrahydrofolate synthetase (*fhs*A), a crucial enzyme of acetogenewsis (Lever et al., 2010), and the gene for the *α* subunit of methyl coenzyme M reductase (*mcrA*), an enzyme that catalyzes the terminal step of biological methanogenesis and is also present in anaerobic methane oxidizers (Friedrich, 2005; Knittel and Boetius, 2009; Wang et al., 2021). Patchy PCR detections of *dsr*AB *and mcr*A in only a few samples (Parkes et al., 2005; Inagaki et al., 2006; Webster et al., 2006) remain at odds with porewater concentration profiles of sulfate and methane, which indicate microbial sulfate reduction, AOM, and methanogenesis (D’Hondt et al., 2004). Similar observations were made based on quantitative PCR and metagenome sequencing in methane-rich deep subseafloor sediments of Hydrate Ridge in the Northeastern Pacific (Colwell et al., 2008), the Black Sea and off Namibia (Schippers et al., 2012), the Baltic Sea (Marshall et al., 2018), and Adélie Basin off Antarctica (Carr et al., 2018). It was thus proposed that methanogens account for low percentages (<1%) of microbial cells in subseafloor sediments, or are not detected by PCR assays due to primer mismatches or use of unrecognized genetic pathways (Lever, 2013).

Here we take a closer look at the *in situ* community of methanogens and anaerobic methanotrophs in the sediment column of ODP Site 1230 in the Peru Trench via PCR assays of *mcrA*. We investigate the relationship between community zonation and geochemical profiles [sulfate, methane, formate, acetate, hydrogen, δ^13^C-methane and -dissolved inorganic carbon (DIC)], and identify active members of the methane-cycling community via reverse transcription-PCR (RT-PCR) of 16S rRNA and *mcrA*-mRNA. Redesigned general *mcrA* primers (Lever and Teske, 2015) and new group-specific *mcrA* and 16S rRNA gene primers allow us to detect methane-cycling functional genes in the AOM and methanogenesis zones inferred from porewater chemical gradients. While updated primers improve the detection of methane-cycling archaea, they reinforce the notion that methane-cycling archaea only account for a small proportion of microbial subsurface communities even in sediments with clear geochemical evidence for methanogenesis and AOM.

## Materials and methods

### Field site and sampling

**Figure 1:**
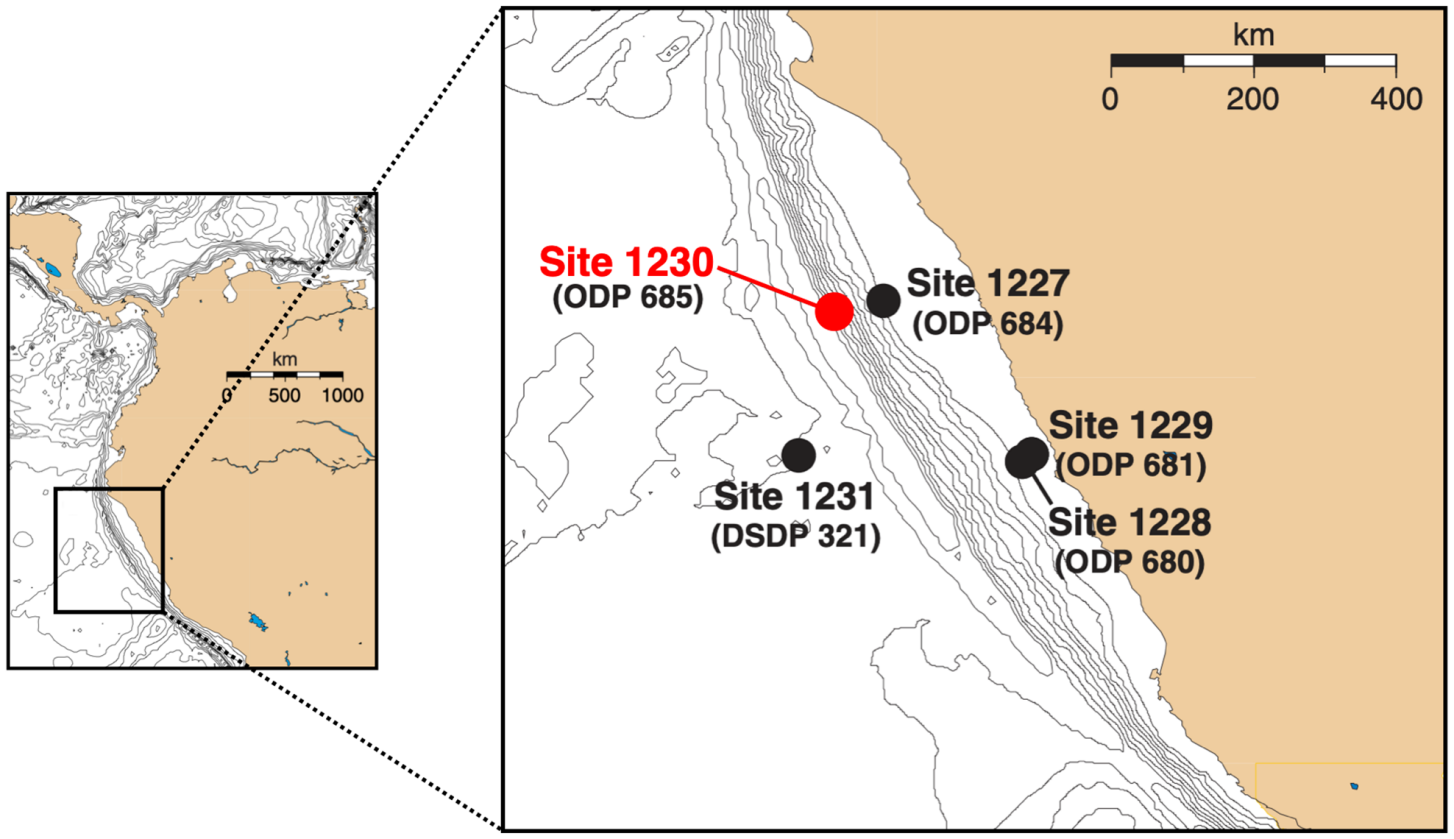
Map of Peru Margin sites sampled during ODP Leg 201 and ODP Leg 112 (in parentheses) [adapted from D’Hondt et al., 2003]. Samples used in this study were collected at ODP Site 1230 in the Peru Trench, which was in the same location as the previously studied ODP Site 685.

The Peru Trench is part of the larger Atacama Trench that is located between the continental South American Plate and the accretionary wedge of the oceanic Nazca Plate (Suess, 1981). ODP Site 1230 is located on the lower slope of the Peru Trench at 5,086 m water depth (Figure 1). Sediments were drilled to ~270 mbsf during ODP Leg 201 in 2002 (D’Hondt et al., 2003). Three boreholes (A, B, and C) were within ~20 m of one another (D’Hondt et al., 2003). Sediment temperatures are low, increasing linearly from 2°C at the seafloor to 12°C at 270 mbsf. The upper 200 m of sediment consist of clay-rich, diatomaceous mud that was largely relocated from the continental shelf throughout the Holocene and Pleistocene. At approximately 216 mbsf, the sediment column changes to Miocene diatom ooze in a stratigraphic hiatus of 4.5 million years (Shipboard Scientific Party, 1988; Meister et al., 2005). Throughout the sediment column, organic carbon contents mostly range from 2 to 4% dry sediment weight (Meister et al., 2005). DIC concentrations to 160 mM and sulfate depletion in the upper ~10 mbsf indicate active microbial remineralization of organic matter, largely by sulfate reduction (D’Hondt et al., 2003). After sulfate is depleted, methane concentrations increase rapidly and reach *in situ* saturation by 28 mbsf (Spivack et al., 2005). Geophysical and chemical data suggest that hydrates are first present at ~70 mbsf and occur intermittently to 278 mbsf (D’Hondt et al., 2003).

For molecular biological analyses, 5-cm whole-round intervals of cores were frozen at −80°C. Only sediment from the nearly contamination-free core interiors was used (House et al., 2003; Lever et al., 2006). For carbon isotope analyses, 5-mL subsamples were frozen in pre-combusted glass vials.

### Porewater geochemical concentrations

**Figure 2:**
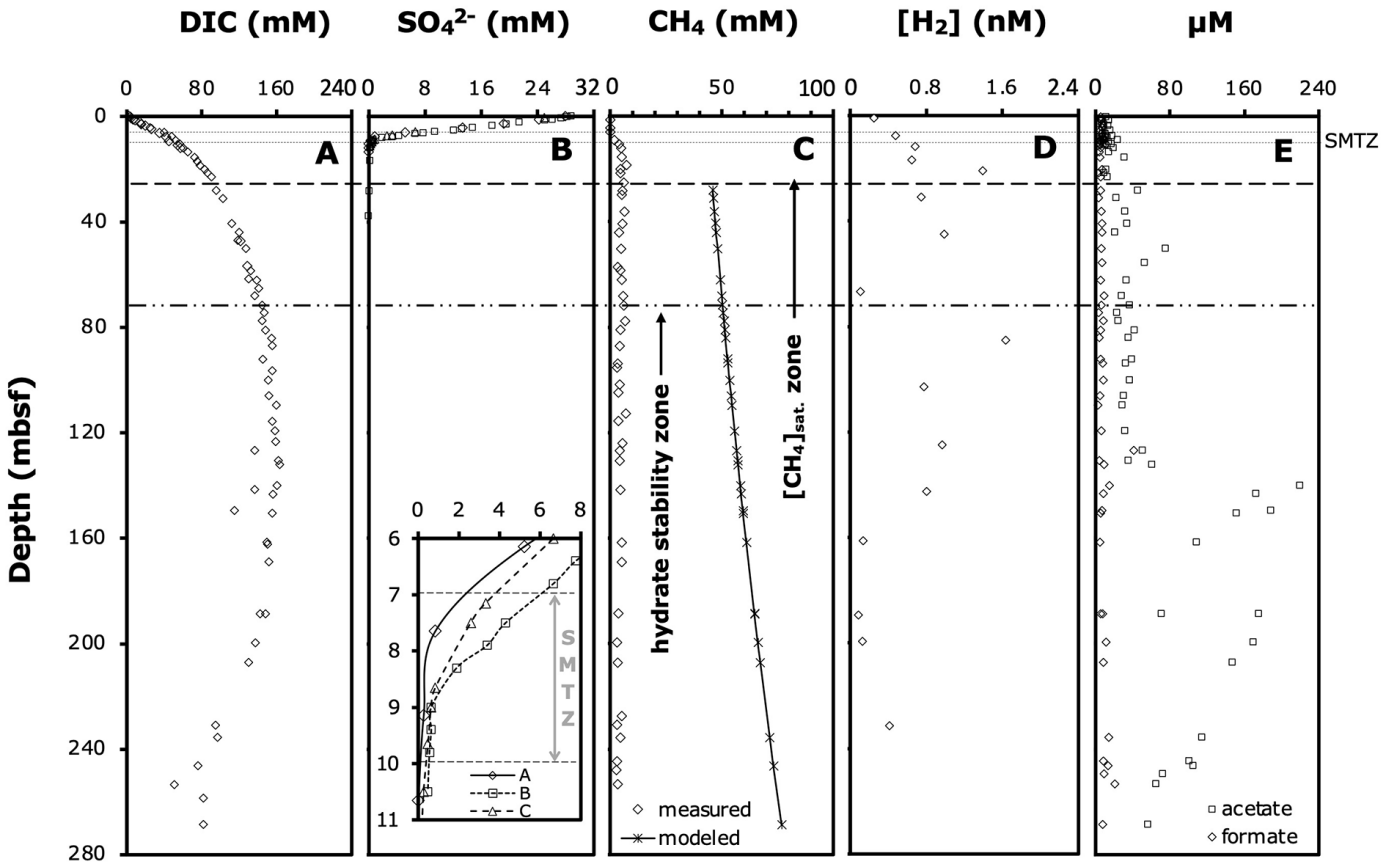
Relevant porewater geochemical profiles: **(A)** DIC concentrations, **(B)** sulfate concentrations (note: the insert shows an enlarged view of the SMTZ and concentrations in individual boreholes), **(C)** measured methane concentrations, modeled methane saturation concentrations, and distribution of hydrate stability zone (D’Hondt et al., 2003) and methane saturation zone (Spivack et al., 2005), **(D)** dihydrogen (H_2_) concentrations, and **(E)** formate and acetate concentrations. Modeled methane concentrations from this study, all other data from D’Hondt et al. (2003). Horizontal lines indicate the approximate depth interval of the SMTZ, and the depths below which we estimate methane concentrations to be saturated and methane hydrates to be present. All data are from Borehole A, except where noted.

We used published depth profiles of DIC, sulfate, methane, dihydrogen (H_2_), formate and acetate concentrations (Figures 2A–E; D’Hondt et al., 2003). Due to outgassing during core retrieval, measured methane concentrations below ~12 mbsf were underestimates of *in situ* concentrations. We calculated methane concentrations below the saturation depth at *in situ* temperature, pressure, and salinity, assuming a uniform pore size of 1.0 μm based on the equilibrium model for methane hydrate-seawater-porous media (Sun and Duan, 2007). Modeled methane concentrations generally agree with measured *in situ* methane concentrations based on pressure coring (4 depths analyzed at ODP Site 1230; Spivack et al., 2005).

### δ^13^C-C_1_ and DIC

**Figure 3:**
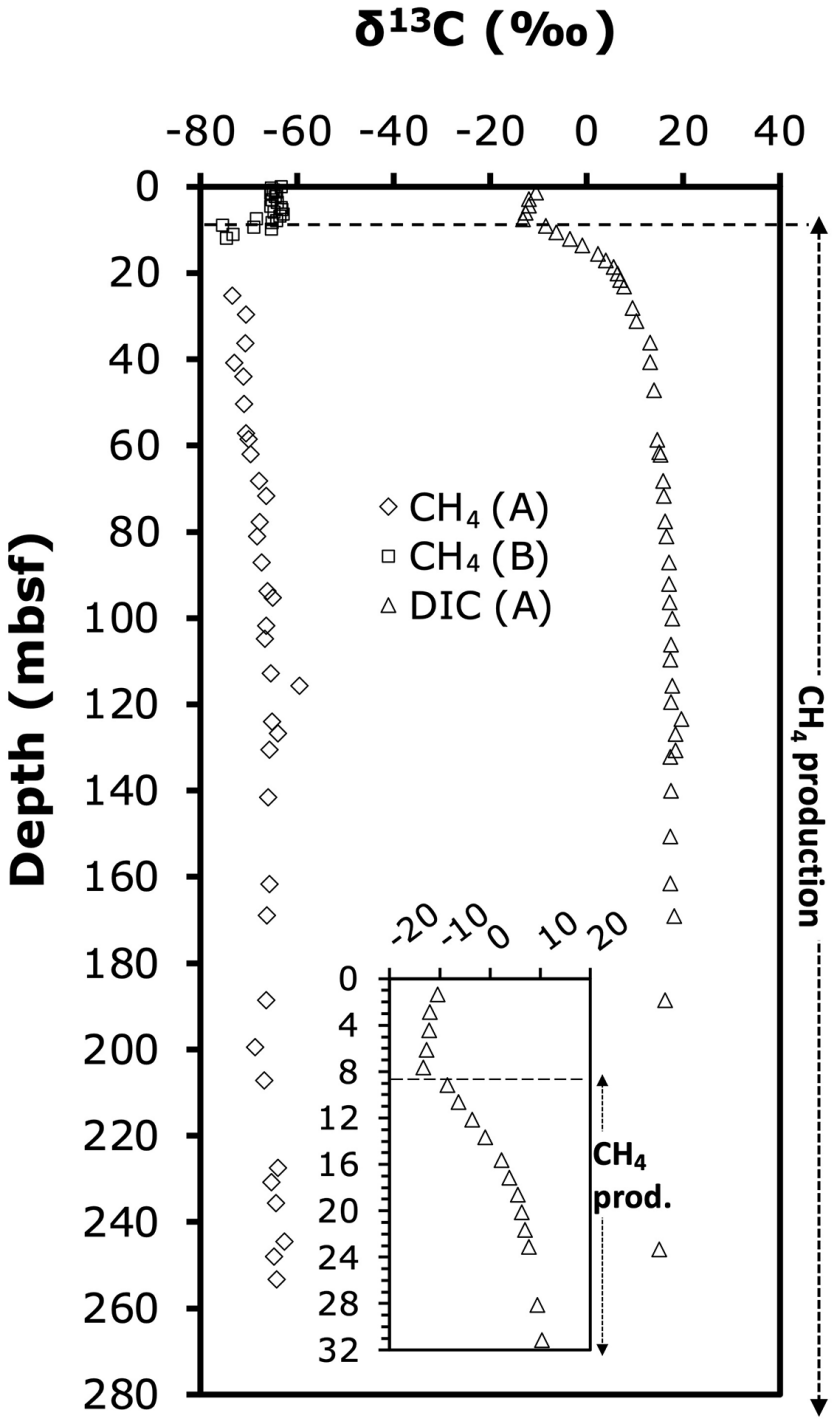
Depth profile of *δ*^13^C-CH_4_ and *δ*^13^C-DIC. Letters in parentheses indicate borehole in which samples were analyzed. Depth profile of difference between *δ*^13^C-CH_4_ and *δ*^13^C-DIC. The dashed line marks the abrupt shift in *δ*^13^C-DIC from decreasing to increasing values.

δ^13^C-C_1_ (~99% ^13^C-CH_4_) and -DIC (Figure 3) were measured as described previously (Biddle et al., 2006). All values are shown in Supplementary Table S1.

### Nucleic acid extraction

RNA was extracted as in Biddle et al. (2006), except that the extraction buffer was supplemented with 120 mM sodium phosphate. DNA was extracted using the same protocol as for RNA, except that the pH of the extraction buffer and phenol were raised to 8.0, the bead beating time reduced to 15 s, and the bead beating speed reduced to 4.0 (Qbiogene, Carlsbad, CA). Moreover, the DNase incubation was omitted, and DNA purified with the PowerClean DNA Clean-Up Kit (MOBIO laboratories, Carlsbad, CA) instead of the RNeasy Mini Kit (Qiagen, Valencia, CA).

### PCR primers

Two previously published general *mcrA* primer pairs yielded no amplification (ME1/ME2, Hales et al., 1996), or amplification at only one depth interval (mcrI, Springer et al., 1995; see Inagaki et al., 2006). The mcrIRD primer pair, a modified version of the mcrI primer pair with a reduced number of nucleotide degeneracies and consequently improved detection sensitivity, was used in conjunction with the ANME-1-specific ANME-1-mcrI primer pair [both published in Lever and Teske (2015)]. Special primers for ANME-1 detection were necessary due to the high number of nucleotide mismatches between the mcrI primer pair primer and the genetically divergent *mcrA* sequences of ANME-1. To confirm that detected *mcr*A detected belonged to active and living members of the methane-cycling community, we performed RT-PCR of *mcrA*-mRNA and 16S rRNA in several depth horizons using new group-specific primers for maximum amplification efficiency and hence detection sensitivity (mRNA: ODP1230, ANME-1; 16S rRNA: Msaeta 268F/927R, ANME-1 42F/898R; ANME-1-SG 35F/1038R). All primer sequences used in this study are shown in Table 1. All nucleotide sequences are publicly accessible at GenBank.

**Table 1:** Overview of PCR primer pairs used in this study.

Gene	Primer pair	Nucleotide sequences (5′-3′)	Reference	Target organisms	T_annealing_ (°C)
*mcrA*	mcrI	F: TAY GAY CAR ATH TGG YT; R: ACR TTC ATN GCR TAR TT	Springer et al. (1995)	General *mcr*A	51
*mcrA*	ME1/ME2	F: GCM ATG CAR ATH GGW ATG TC; R: TCA TKG CRT AGT TDG GRT AGT	Hales et al. (1996)	General *mcr*A	58
*mcrA*	*mcrIRD*	F: TWY GAC CAR ATM TGG YT; R: ACR TTC ATB GCR TAR TT	Lever and Teske (2015)	General *mcr*A	55
*mcrA*	*ANME-1-mcrI*	F: GAC CAG TTG TGG TTC GGA AC; R: ATC TCG AAT GGC ATT CCC TC	Lever and Teske (2015)	ANME-1 *mcr*A	63
*mcrA*	ODP1230-mcrI	F: GCT ACA TGT CCG GTG G; R: CGG ATA GTT GGG TCC TCT	This study	ODP 1230 *M.thrix*	59
16S	M.saeta 268F/927R	F: CCT ACT AGC CTA CGA CGG GT; R: CCC GCC AAT TCC TTT AAG TTT	This study	All *Methanothrix*	63
16S	ANME-1 42F/898R	F: GAG TTC GAT TAA GCC ATG TTA GT; R: CGA CCG TAC TCC CCA GAT	This study	ANME-1a-b	61
16S	ANME-1-SG 35F/1038R	F: GCT ATC AGC GTC CGA CTA AGC; R: TAA TCC GGC AGG GTC TTC A	This study	ANME-1d	65
16S	ARC 8F/915R	F: TCC GGT TGA TCC TGC C; R: GTG CTC CCC CGC CAA TTC CT	Stahl and Amann (1991)	All Archaea	55

PCR assays involving the universal ARC 8F/915R primer pair were solely used to confirm the recovery of PCR-amplifiable nucleic acids from Archaea in all samples based on gel electrophoresis images of PCR products.

### PCR protocols

PCR assays of *mcr*A were performed using the Takara SpeedSTAR HS DNA polymerase kit (TaKaRa Bio USA, Madison, WI) using (1) 1 × 2 min denaturation (98°C), (2) 40 × (a) 10s denaturation (98°C), (b) 30s annealing (Table 1 for temperatures), (c) 1 min extension (72°C), and (3) 1 × 5 min extension (72°C). Negative controls and reaction blanks were included.

RT-PCR assays were carried out using TaKaRa RNA PCR Kits (AMV) Version 3.0 (TaKaRa Bio USA, Madison, WI) and (1) 1 × 15 min reverse transcription, (2) 5 min denaturation (98°C), (3) 40 × (a) 30s denaturation (98°C), (b) 30s annealing (Table 1 for temperatures), (c) 1 min extension (72°C), and (4) 1 × 5 min extension (72°C). Negative controls and reaction blanks were included. Absence of DNA was confirmed by DNA-PCR with the same treatments but omitting the reverse transcription step.

### Cloning and sequencing

PCR products were purified in a 2.5% low-melting point agarose gel using 1 × Tris acetate - EDTA buffer (TAE). Gel slices containing PCR fragments of the correct length were excised and purified using a S.N.A.P. Mini Kit (Invitrogen, Carlsbad, USA). Purified PCR fragments were cloned using the Topo TA Kit (Invitrogen, Carlsbad, USA) and transformed into TOP10 electrocompetent cells following the manufacturer’s instructions. Plasmid extraction and purification was done using the GeneJET Plasmid Miniprep Kit (ThermoFisher Scientific) and cycle sequencing was performed on an ABI 3730 Sequencer with M13 universal primers (SP010-SP030) at the Josephine Bay Paul Center at MBL (Woods Hole, MA). Sequences were BLAST analyzed using the nucleotide collection in GenBank.^1^ Phylogenetic trees were created and bootstrap analyses (1,000 replicates) performed in ARB^2^ using manually optimized SILVA 16S rRNA gene alignments, and a custom-built, publicly accessible *mcr*A database (name: mcrA4All)^3^ with >2,400 high-quality, aligned *mcr*A amplicon and genome sequences.

### Thermodynamic calculations

Gibbs energy yields (Δ*G_r_*) of methanogenesis reactions from H_2_ + CO_2_ (2 HCO_3_^−^ + 4 H_2_ + H^+^ ➔ CH_4_ + 3 H_2_O) and acetate (CH_3_COO^−^ + H_2_O ➔ CH_4_ + HCO_3_^−^), and the methanogenic conversion of formate to methane [4 HCOO^−^ + H_2_O + H^+^ ➔ CH_4_ + 3 HCO_3_^−^; note: this reaction presumably involves the initial oxidation of formate to H_2_ and HCO_3_^−^, which is not known to conserve energy except in certain hyperthermophiles (Schink et al., 2017)] were calculated based on the equation:

$$\Delta G_{r}=\Delta G_{r}^{0}+RT\ln Q_{r}$$

where ΔG*_r_*^0^ is the Gibbs energy (kJ mol^−1^ of reaction) at standard concentrations (1 M for reactants and products, pH 7.0), corrected for *in situ* temperature T (K) and pressure *p* (bar) based on standard enthalpies and molar volumes as outlined in Stumm and Morgan (1996), R is the universal gas constant (0.008314 kJ mol^−1^ K^−1^), and *Q_r_* the quotient of product and reactant activities. Calculations were done for measured pH and concentrations of DIC (HCO_3_^−^), H_2_, and acetate. Measured methane concentrations were used for the upper 12 mbsf, while modeled concentrations were used below. Activities of all chemical species were calculated by multiplying concentrations by their activity coefficients. These were γ_HCO32−_ = 0.532 (Millero and Schreiber, 1983), and γ_CH4_ = 1.24 (Millero, 2000). The activity coefficients of H_2_, acetate, and formate were approximated with those of CH_4_ (H_2_) and HCO_3_^−^ (acetate, formate). Standard Gibbs energies (∆G*_f_* °), standard enthalpies (∆H*_f_* °), and standard molal volumes (∆V*_f_* °) of formation are shown in Supplementary Table S2.

## Results

Porewater gradients of chemical species determined on ODP Leg 201 provided the initial framework for our study and indicated ODP Site 1230 as a deep-sea site with unusually organic-rich sediments and highly active anaerobic microbial communities. Organic matter remineralization by microbes to at least 140 mbsf was indicated by DIC concentrations that increased steeply in the upper 25 mbsf and continued to increase gradually to 140 mbsf (Figure 2A). Sulfate reducing microbial communities depleted sulfate at ~9 mbsf in borehole A and up to 1 m deeper in boreholes B and C (Figure 2B). Porewater methane concentrations in borehole A were at background values (0.06 mM) at 6.10 mbsf, but had increased to 1.86 mM at 9.1 mbsf (Figure 2C). We thus estimate that the sulfate–methane transition zone (SMTZ), where most AOM takes place, was located within the depth interval from 7 to 9 mbsf in borehole A and up to 1 m deeper in boreholes B and C (Figure 2B, insert; for enlarged view of sulfate and methane profiles across the SMTZ in borehole A, see Supplementary Figure S1). Below the SMTZ, methane concentrations increased steeply, reaching saturation by ~28 mbsf, and hydrates appeared by ~50 mbsf. Hydrogen (H_2_) concentrations fluctuated greatly, but generally increased throughout the sulfate reduction zone, stabilized in the methanogenesis zone to ~140 mbsf, and decreased below (Figure 2D). Formate concentrations showed no clear depth-related trend and fluctuated between 3–15 μM throughout the entire cored interval (Figure 2E). By contrast, acetate concentrations increased from 3–11 μM in the sulfate reduction zone and SMTZ (upper 10 mbsf) to concentrations of ~20–60 μM in the methanogenesis zone between 30 to 140 mbsf (Figure 2E; see Supplementary Figure S2 for enlarged view of upper 20 mbsf). Below 140 mbsf, acetate concentrations rose sharply to 220 μM and remained >50 μM to the deepest cores sampled.

### Carbon isotope geochemistry

^13^C-isotopic signatures of porewater methane and DIC provide insights into the zones of biological methane production and oxidation (Figure 3). Throughout the sediment column, methane was ^13^C-depleted relative to DIC. *δ*^13^C-DIC-values (only determined in borehole A) decreased slightly from −10.4‰ in the upper meter to −13.3‰ by 7.65 mbsf, then increased sharply in the uppermost methanogenic layer to +6‰ at 20 mbsf. The steepest increase in *δ*^13^C-DIC occurred within the interval from 7.65 mbsf (*δ*^13^C-DIC: −13.2‰) to 9.15 mbsf (*δ*^13^C-DIC: −8.4‰), and suggests onset of methanogenesis by CO_2_ reduction in this interval. Below 20 mbsf, *δ*^13^C-DIC-values continued to gradually increase to reach a maximum of +20‰ at 123 mbsf, below which values slightly fell off to +15‰ at 246 mbsf (Figure 3). The ^13^C-isotopic compositions of methane, determined in the methanogenesis zones of boreholes A (>25 mbsf) and B (0–12 mbsf), were in a range typical of biological methanogenesis (Whiticar et al., 1986; Whiticar, 1999)*. δ*^13^C-CH_4_ was ~ −65‰ in the upper 6 mbsf, and then decreased to −75‰ at 12 mbsf. This increase in *δ*^13^C-CH_4_ upward through the SMTZ is consistent with isotopic discrimination of AOM against ^13^C-CH_4_. Below, values gradually increased from to ~ −65‰ at 246 mbsf (Figure 3). The difference in *δ*^13^C-CH_4_ relative to *δ*^13^C-DIC was ~ −53‰ in the upper 6 mbsf, decreased across the SMTZ reaching −71‰ in the upper methanogenesis zone at 12 mbsf, and stabilized at −80‰ to −85‰ below 25 mbsf (Figure 3).

### *mcr*A sequence diversity

**Figure 4:**
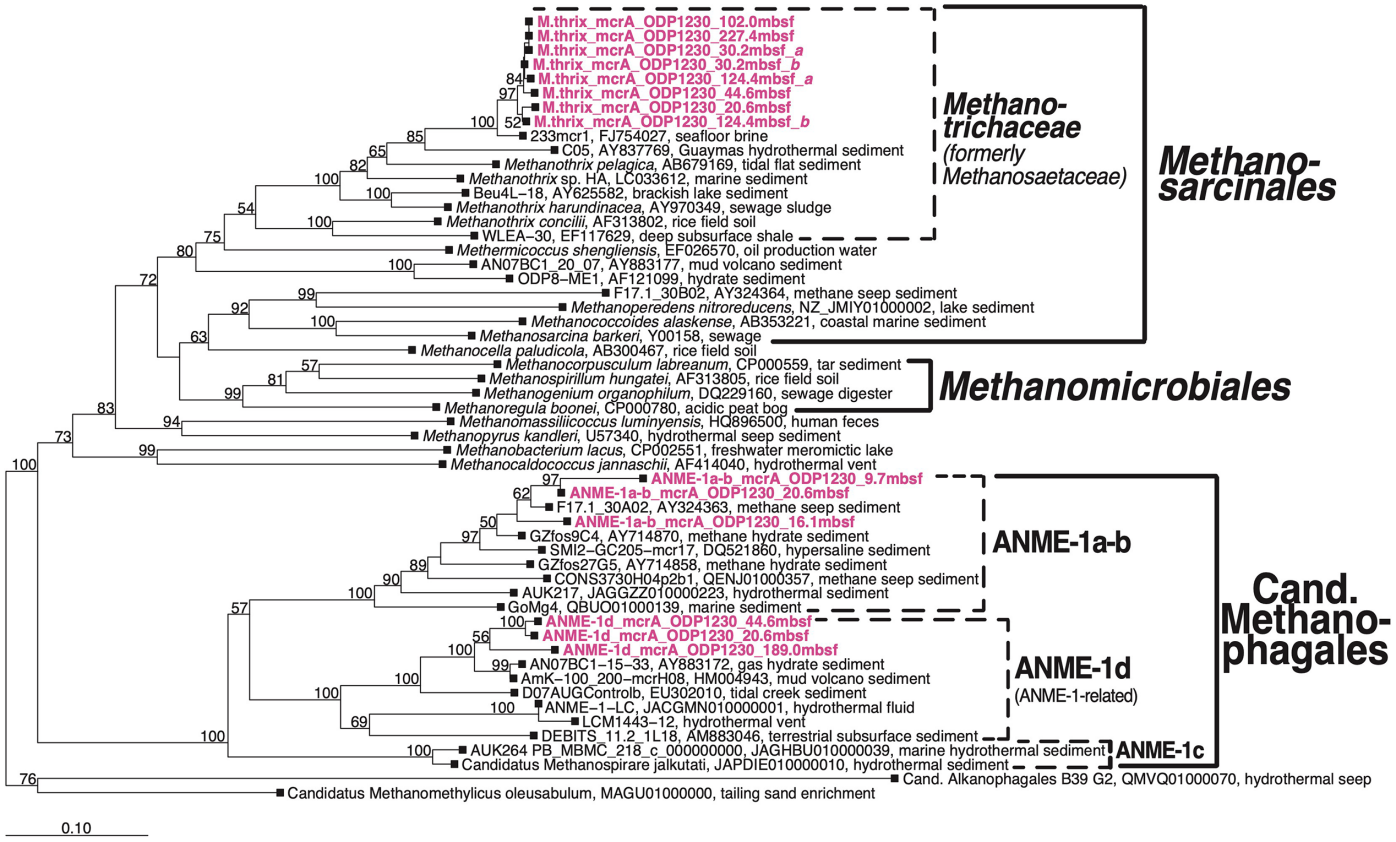
Bootstrap phylogenetic tree based on *mcrA* nucleotide sequences. Created using Jukes-Cantor correction in ARB neighbor-joining (Ludwig et al., 2004). Cand., Candidatus.

We detected *mcrA* sequences of three phylogenetic groups (Figure 4): (1) putatively anaerobic methanotrophic ANME-1a-b Archaea, (2) a sister group of ANME-1, which we here refer to as ANME-1d, and (3) sequences of *Methanotrichales* that cluster with a genus-level group that includes the known aceticlastic methanogens *Methanothrix harundinacea* and *Methanothrix pelagica*.

**Figure 5:**
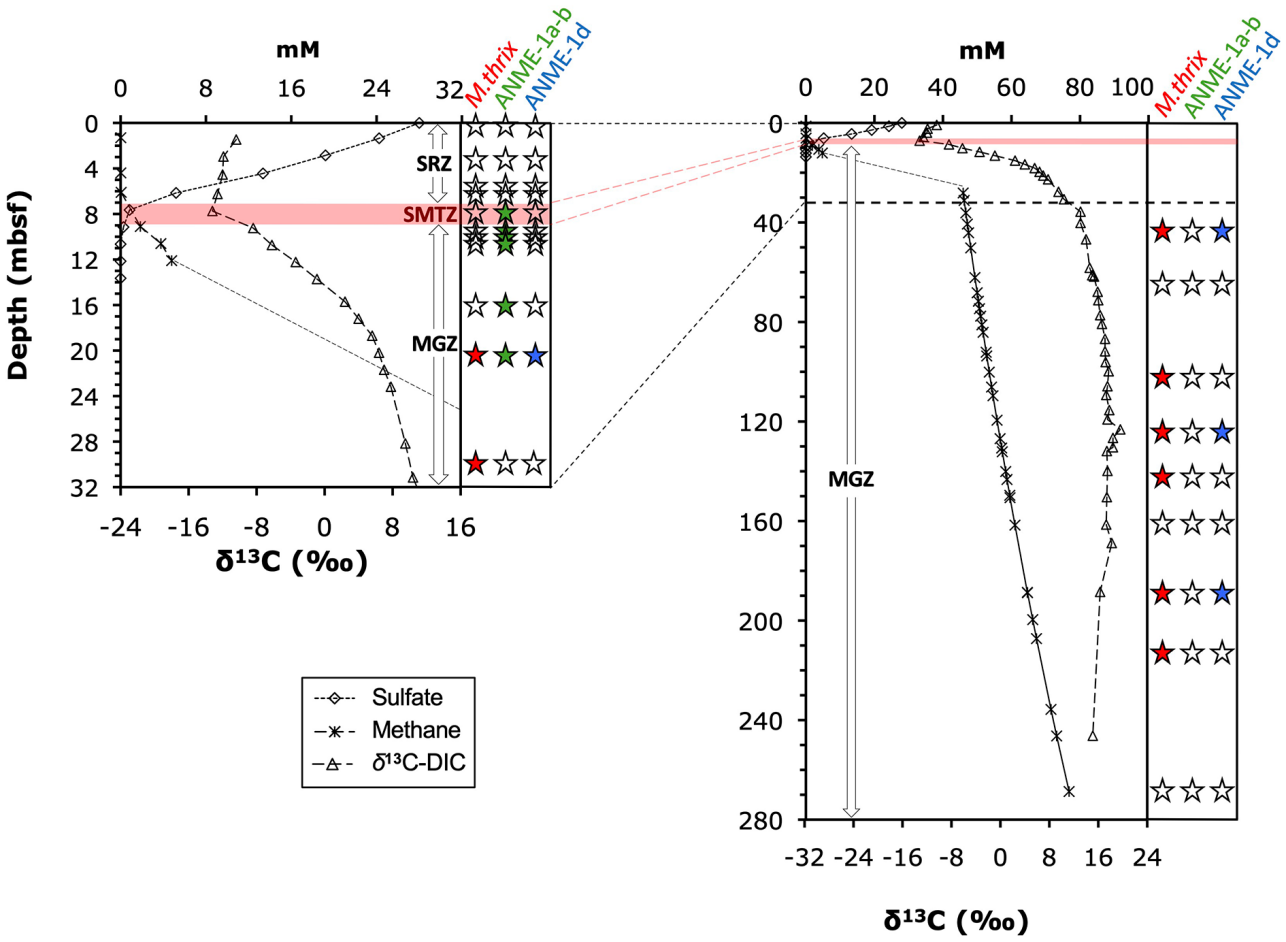
Distribution of *mcr*A groups along depth and geochemical gradients of sulfate, methane, and *δ*^13^C-DIC. Panel on right side of each graph indicates detection/absence of detection of (1) ANME-1a-b, (2) ANME-1d, and (3) *Methanothrix* sequences. Solid black symbols indicate detection, empty symbols indicate lack of detection (example: 
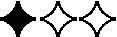
 indicates presence of ANME-1, and absence of ANME-1d and *Methanothrix*). Horizontal red bar indicates the depth interval of the SMTZ, where most AOM takes place, for Borehole A (7 to 9 mbsf). This interval extended ~1 m deeper (~10 mbsf) in Borehole B. The uppermost detections of ANME1-a-b *mcr*A were at 7.8 and 9.7 mbsf in Borehole B and thus near the upper and lower limits of the SMTZ in this borehole.

The three groups were vertically zonated (Figure 5). ANME-1a-b *mcrA* sequences were found in horizons near the upper (7.8 mbsf) and lower limit (9.7 mbsf) of the STMZ in Borehole B and four horizons in the upper methanogenesis zone (10.25–20.6 mbsf; Table 2). Sequences of ANME-1d were detected with ANME-1a-b sequences at one depth in the upper methanogenesis zone (20.6 mbsf) and in three horizons below (to 189.0 mbsf). *Methanothrix mcr*A showed a distribution similar to ANME-1d, but was detected in more sediment horizons and to greater depth (to 227 mbsf; Table 2).

**Table 2:** Overview of boreholes, core samples, sediment depths, and biogeochemical zones from which DNA and RNA were extracted, and the results of PCR amplifications with different primers with number of clones sequenced in parentheses.

Bore-hole	Core, section, interval (cm)	Depth (mbsf)	Biogeo-chemical zone	DNA			mRNA	16S rRNA
				*mcr*IRD	ODP1230_ *M.thrix-mcr*A	ANME-1-*mcr*I-DNA	ANME-1-*mcr*I-mRNA	*M.thrix*-16S-rRNA-268F/927R
A	1H-1, 25–30	0.3	SRZ	bd	-	bd	-	-
A	1H-3, 25–30	3.3	SRZ	bd	-	bd	-	-
B	2H-2, 120–125	5.70	SRZ	bd	-	bd	bd	-
B	2H-3, 30–40	6.30	SRZ	bd	-	bd	bd	-
B	2H-4, 30–40	7.80	SMTZ	bd	-	ANME-1a-b	bd*	-
B	2H-5, 70–80	9.70	SMTZ (MGZ?)	bd	-	ANME-1a-b	bd*	-
B	2H-5, 120–125	10.20	MGZ	bd	-	ANME-1a-b	bd*	-
C	2H-5, 25–30	10.8	MGZ	bd	-	ANME-1a-b	bd*	-
A	3H-2, 25–30	16.1	MGZ	bd	-	ANME-1a-b (45)	-	-
A	3H-5, 25–30	20.6	MGZ	M.thrix (17)	-	ANME-1a-b (1), ANME-1d (40)	-	-
A	4H-5, 35–40	30.2	MGZ	M.thrix (28)	-	bd	-	-
A	6H-2, 25–30	44.6	MGZ	M.thrix (9)	-	ANME-1d (40)	ANME-1d (3)	M.thrix (7)
A	9H-5, 23–28	65.7	MGZ	bd	bd	bd	-	-
A	13H-3, 20–25	102.0	MGZ	M.thrix (30)	-	bd	-	-
A	15H-6, 25–30	124.4	MGZ	bd	M.thrix (19)	ANME-1d (47)	bd	M.thrix (12)
A	18H-3, 35–40	142.2	MGZ	bd	M.thrix (20)	bd	-	-
A	21H-3, 25–30	160.5	MGZ	bd	-	bd	-	-
A	24H-2, 24–29	189.0	MGZ	M.thrix (25)	-	ANME-1d (44)	-	-
A	30X-1, 108–115	227.4	MGZ	M.thrix (30)	-	bd	bd	bd
A	38X-1, 130–135	268.5	MGZ	bd	bd	-	-	-

SRZ, sulfate reduction zone; SMTZ, sulfate–methane transition zone; MGZ, methanogenesis zone; bd, below PCR detection; −, not tested; M.thrix, Methanothrix [we did not detect mcrA-mRNA using the general mcrIRD primer pair].

We detected *mcr*A-mRNA of ANME-1-d in one of the 9 samples examined using the ANME-1-*mcr*A primer pair (core 6H-2, 44 mbsf; Table 2). In 11 replicate RT-PCRs of RNA extracts, controls (PCR negative, extraction blank, DNA controls) always tested negative, whereas 8/11 RNA extracts tested positive. By comparison, four other samples (from 7.8, 9.7, 10.2, 10.8 mbsf) yielded RT-PCR detection with the same primers, but DNA controls were positive (albeit weaker than cDNA bands), indicating that traces of DNA had resisted the DNAse treatment. We cloned cDNA of *mcr*A-mRNA from core 6H-2 and confirmed the presence of *mcr*A of ANME-1d.

In addition to *mcr*A-mRNA, we examined 16S rRNA sequences. RT-PCRs with new *Methanothrix*-specific 16S rRNA gene primers (Table 1) yielded *Methanothrix*-like sequences in two additional depth horizons (Table 2; Supplementary Figure S3). This primer pair also generated 16S rRNA sequences of a sister group of *Methanosarcinales*, previously detected in methane seep and mud volcano environments, with unknown metabolism at two depths (15H-6, 30H-1; Supplementary Figure S3). Interestingly, despite detecting mRNA with ANME-1-*mcr*A primers, we were unable to detect 16S rRNA of ANME-1a-b or ANME-1d with newly designed group-specific 16S rRNA gene primers (Table 1).

## Discussion

We present a depth profile of *mcrA* that relates distribution patterns of deep subseafloor methanogens and anaerobic methanotrophs to the geochemical context. While the genetic and gene transcript analyses in our study are present-day snapshots of methane-cycling activity, the measured geochemical data in part capture much longer time scales, such as the accumulation of methane over millions of years. Nonetheless, we observe a clear relationship between the community profile of methane-cycling archaea and porewater geochemical gradients. We, moreover, resolve the paradox of earlier studies in which methane-rich sediments at ODP Site 1230 appeared largely devoid of methanogens in the methanogenesis zone (Inagaki et al., 2006), and completely devoid of anaerobic methanotrophs in the SMTZ (Biddle et al., 2006).

Geochemical and functional gene profiles indicate a distinct depth stratification of the active methane cycling community (Table 2; Figure 5). No nucleic acid evidence of present-day methane-cycling was detected in the upper part of the sulfate reduction zone (0 to ~7 mbsf) despite methane concentrations in the micromolar range. Throughout the SMTZ (~7 to 9 mbsf in borehole A, up to 1 m deeper in boreholes B and C), sulfate concentrations diminished in typical concave-down profiles, and methane concentrations increased. These gradients coincide with the detection of *mcr*A of ANME-1a-b (Figure 5), members of which are known to be anaerobic methanotrophs (Knittel and Boetius, 2009). Therefore, our sulfate and methane concentration profiles and *mcrA* composition in the SMTZ are consistent with AOM. In the underlying methanogenesis zone (~9 to 269+ mbsf), methane concentrations and *δ*^13^C-DIC increase drastically in the upper tens of meters and stay high throughout, while sulfate remains depleted (Figures 2, 3). Interestingly, ANME-1a-b Archaea were detected in the upper meters of the methanogenesis zone (from 9.7 to 16.1 mbsf), in line with past indications that ANME-1a-b might be capable of methanogenesis in addition to methanotrophy (House et al., 2009; Lloyd et al., 2011; Beulig et al., 2019). Moreover, ANME-1a-b were vertically separated from ANME-1d and *Methanothrix mcr*A sequences, which were only found in deeper, methanogenic sediment layers. The three groups only overlapped in core 3H-5 (20.6 mbsf), which marked the deepest sample in which ANME-1a-b and shallowest sample in which ANME-1d and *Methanothrix* were detected.

### Implications of the ^13^C-isotopic data

The changes in *δ*^13^C-DIC and –methane provide insights into the sources of DIC and pathways of methane production at ODP Site 1230. The *δ*^13^C-DIC isotopic values in the upper ~9 mbsf (−10.4 to −13.2‰) are consistent with organic matter mineralization becoming the main DIC source with increasing sediment depth. Most of this organic matter is likely to be phytoplankton-derived organic matter (*δ*^13^C-total organic carbon: ~22–23‰; Biddle et al., 2006) that was initially deposited under the upwelling regime of the Peru Margin, and subsequently reworked and laterally transported downslope to the Peru Trench. Toward the sediment surface, the *δ*^13^C-DIC increases, most likely due to an increasing contribution of ^13^C-enriched DIC from deep sea bottom water, which typically bears a ^13^C-composition of ~0 to +1.2‰ (Lynch-Stieglitz et al., 1995).

Notably, despite the strong geochemical evidence for AOM in the SMTZ, which might be expected to produce highly ^13^C-depleted DIC from the oxidation of methane, we do not observe a strong downward swing in *δ*^13^C-DIC within the SMTZ. This phenomenon has been observed previously in SMTZs and has been explained with concomitant AOM and methane production (Beulig et al., 2019), microbially mediated isotope exchange between methane and DIC (Yoshinaga et al., 2014), and reversibility of intracellular methane-cycling reactions at low sulfate concentrations (Wegener et al., 2021). In our case, the *mcr*A data argue against the first scenario, if ANME-1a-b are assumed to only perform methanotrophy. Yet, if – as proposed previously - ANME-1a-b are facultative methanogens, which matches the detection of this group throughout the upper ~12 m of the methanogenesis zone, then the first scenario is also plausible. Notably, porewater dissolved barium concentrations increase sharply throughout the AOM and upper methanogenesis zone (e.g., from 2.7 μM at 6.15 mbsf to 290 μM at 23.15 mbsf at ODP Site 1230A; D’Hondt et al., 2003), consistent with (slow) release of sulfate through chemical dissolution of barite (BaSO_4_). This sulfate could fuel low rates of AOM, and thus also support concomitant AOM and methane production throughout the upper methanogenesis zone. AOM coupled to iron or manganese reduction could also support low rates of AOM, as was recently proposed for subsurface sediments of the South China Sea, where ANME-1 were detected meters below the SMTZ (Zhang et al., 2023). Yet, the low porewater concentrations of Fe^2+^ (0.6 to 3.9 μM) and Mn^2+^(0 to 0.3 μM) in the upper methanogenic sediment layer where we detected ANME-1a-b at ODP Site 1230 (D’Hondt et al., 2003) do not support an important role of AOM coupled to metal reduction.

**Figure 6:**
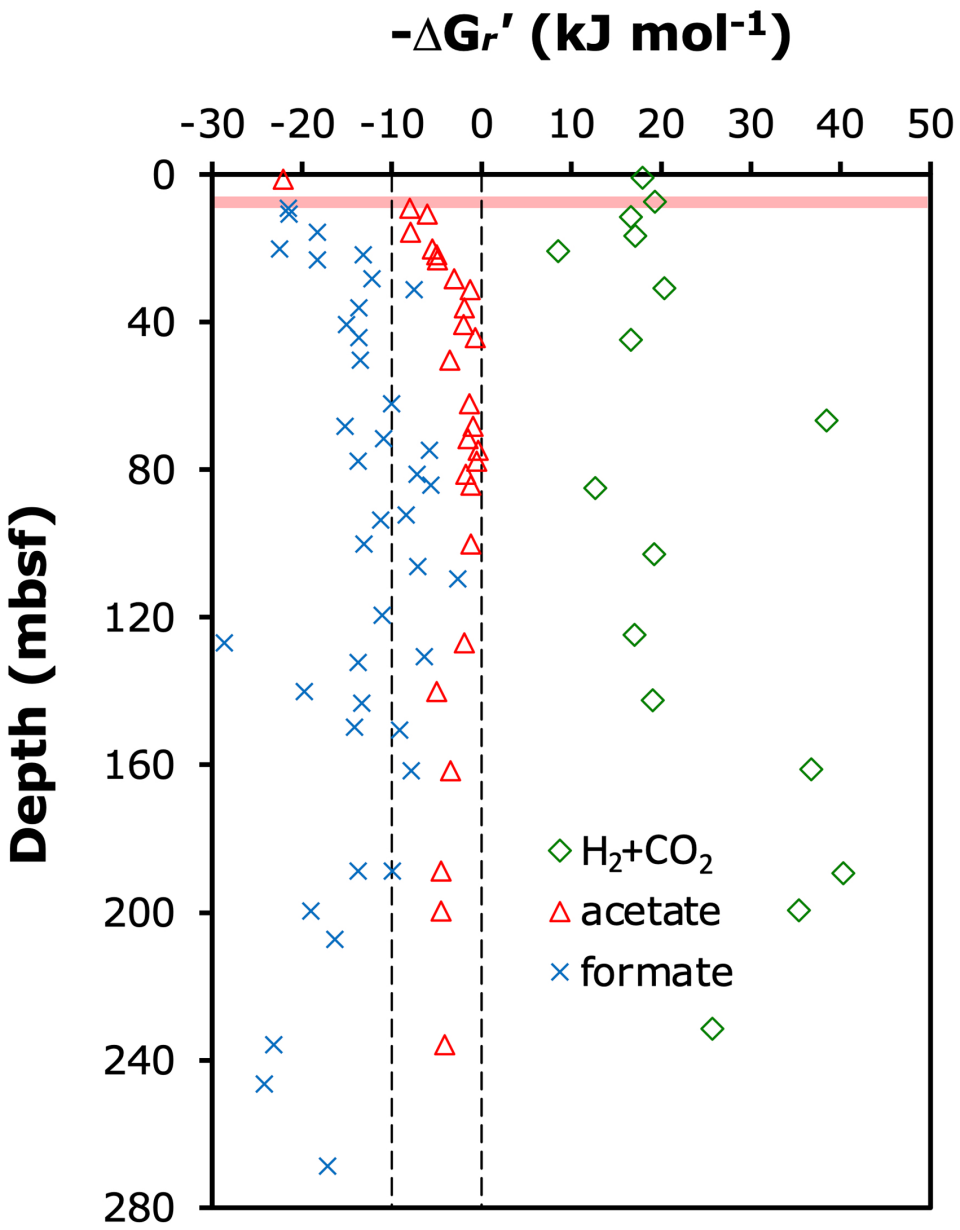
Calculated *in situ* Gibbs energy yields (Δ*Gr*’) of methanogenesis reactions from H2  +  CO2 (HCO3^–^ +  4 H2  +  H^^+^^ ➔ CH__4_ _ +  3 H_2_O) and acetate (CH_3_COO^–^ + H_2_O ➔ CH_4_ + HCO_3_^–^), and the methanogenic conversion of formate to methane (4 HCOO^–^ + H_2_O + H^+^ ➔ CH_4_ + 3 HCO_3_^–^; note: this reaction includes the initial oxidation of formate to H_2_ and HCO_3_^–^, which may not be coupled to energy conservation). Calculations were done for *in situ* conditions as described in the Materials & Methods.

Below 9 mbsf, the *δ*^13^C-DIC increased, consistent with a strong isotopic imprint of methanogenesis by CO_2_ reduction. Strong isotopic discrimination against ^13^C-CO_2_ is the norm in methanogenesis from H_2_/CO_2_ (Whiticar, 1999; Penning et al., 2005) and can result in significant ^13^C-enrichment of the residual DIC pool (Alperin and Hoehler, 2009; House et al., 2009). Based on measured porewater geochemical data, methanogenesis from H_2_/CO_2_ is, however, not thermodynamically favorable (Figure 6), with *in situ* Gibbs energies in the positive (i.e., endergonic) range (ΔG*_r_*’ > 0 kJ mol^−1^) throughout the sediment column of ODP Site 1230. Since methanogenesis from formate follows the same biochemical route as hydrogenotrophic methanogenesis after the initial oxidation of formate to CO_2_ and H_2_ by formate dehydrogenase (Sparling and Daniels, 1986), a similar isotopic fractionation can be expected. Indeed, the complete conversion reaction of formate to methane is thermodynamically favorable, and based on that alone formate a potential methanogenic substrate at ODP Site 1230 (Figure 6). Yet, assuming that energy is not conserved during the initial formate oxidation step, but only in the second step involving methanogenic CO2 reduction with H_2_ (Schink et al., 2017), then formate conversion to methane appears less plausible. This is because intracellular H_2_ concentrations can be expected to be close to equilibrium with H_2_ concentrations in the surrounding sediment due to H_2_ leakage out of methanogenic cells (Finke et al., 2007). As stated above, however, measured H_2_ concentrations in the surrounding sediment are too low to energetically support hydrogenotrophic methanogenesis. A more recently documented form of methanogenic CO_2_ reduction involves interspecies electron transfer (IET). This form of methanogenesis, which was first discovered in *Methanothrix harundinaceae* (Rotaru et al., 2014), involves cellular structures, e.g., cytochromes, that attach to conductive mineral surfaces or syntrophic partner organisms (Gao and Lu, 2021). The isotopic fractionations of these reactions are not known but most likely also cause *δ*^13^C-enrichment of residual DIC. In principle, the conversion of formate to methane could also operate via a direct electron transfer mechanism, e.g., from syntrophic bacteria to methanogens. This mechanism could bypass H_2_ as a catabolic intermediate and even render formate catabolism a potential source of methane. Thus, based on the available geochemical data, the dominance of methanogenic CO_2_ reduction at Site 1230, which was inferred from the *δ*^13^C-DIC profile below 9 mbsf, is most plausibly explained with electron transfer from syntrophic partner organisms or mineral surfaces to methanogens.

By contrast, chemoautotrophy, acetogenesis or other methanogenic pathways are unlikely drivers of the observed *δ*^13^C-DIC increase in the methanogenesis zone. Although (certain) ANME-1a-b are chemoautotrophs (Kellermann et al., 2012), past studies indicate that AOM of isotopically highly depleted methane (~ − 75 per mil) to CO_2_ occurs at much (≥40-fold) higher rates than C-assimilation by chemoautotrophy (e.g., Nauhaus et al., 2007; Wegener et al., 2008). Consequently, AOM would be expected to overprint any C-isotopic enrichment of DIC by chemoautotrophy. Acetogenesis from H_2_/CO_2_, which also strongly discriminates against ^13^C (Gelwicks et al., 1989), is unlikely based on *δ*^13^C-acetate values of −12 to −18‰ at ODP Site 1230 that indicate fermentation as the main acetate source (Heuer et al., 2006). The other widespread methanogenesis pathways from acetate (aceticlastic methanogenesis) and methylated substrates (e.g., methanol, dimethyl sulfide and methyl amines; methylotrophic methanogenesis), produce rather than consume CO_2_ (Whitman et al., 2014). In aceticlastic methanogenesis, this CO_2_ has the same ^13^C-depleted isotopic composition as the methane produced (Gelwicks et al., 1994) and would thus lower (rather than increase) the *δ*^13^C-DIC. Despite high concentrations of acetate, our calculations, moreover, indicate that aceticlastic methanogenesis is close to thermodynamic equilibrium throughout most of the methanogenesis zone (Figure 6), with Gibbs energies not reaching the theoretical minimum required for biological energy conservation by proton translocation (ΔG*_r_*’ ≅ 10 kJ mol^−1^; Hoehler et al., 2001; Lever et al., 2015). ^13^C-depletion of DIC is also expected for methylotrophic methanogenesis, even though this pathway produces methane with similar isotopic fractionations as CO_2_ reduction (Conrad, 2005). The reason for ^13^C-depletion of DIC is that the main isotopic fractionation of methylotrophic methanogenesis is produced by the first enzymes in the reaction chain (methyl transferase I and/or II; Krzycki et al., 1987) and hence upstream of where C fractions enter separate enzymatic pathways to produce CO_2_ and methane (Thauer, 1998). Notably, another form of methylotrophic methanogenesis, which involves methylated substrates and hydrogen, e.g., methanol + H_2_ does not produce CO_2_ (Dridi et al., 2012; Whitman et al., 2014). A fourth methanogenic pathway that involves the conversion of methoxy-groups from lignin monomers to methane with CO_2_ as a co-substrate (Mayumi et al., 2016) is in theory also possible. Yet, this pathway is unlikely to be important given the primarily phytoplanktonic origin of organic matter at ODP Site 1230 (Shipboard Scientific Party, 1988; D’Hondt et al., 2003) and recent evidence suggesting minimal long-term degradation of lignin in anoxic sediment (Han et al., 2022).

The increase in *δ*^13^C-DIC with depth is steepest from the lower SMTZ to ~20 mbsf (Figures 3, 5), consistent with rates of methanogenic CO_2_ reduction being highest in this interval. The subsequent decrease in the slope of *δ*^13^C-DIC with depth can be explained with an increase in the DIC pool size and decline in the rates of CO_2_ reduction. In addition, it is possible that the relative contributions of other methanogenic pathways, e.g., aceticlastic methanogenesis, increase below this depth. Nonetheless, the ^13^C-isotopic depletions of −80 to −86‰ of *δ*^13^C-methane relative to *δ*^13^C-DIC that were consistently measured below 18 mbsf (Figure 3) indicate that CO_2_ reduction accounts for most of the methane that has accumulated throughout the methanogenesis zone of ODP Site 1230.

### Community zonation

When examined in the geochemical context, the distribution of *mcrA* genes within the methanogenesis zone of ODP Site 1230 may be surprising. Isotopic compositions of DIC and methane suggest predominance of methanogenesis by CO_2_ reduction, whereas detected *mcr*A sequences belong to phylotypes of putatively anaerobic methane-oxidizing ANME-1a-b, its catabolically uncharacterized sister group ANME-1d, and *Methanothrix*, a genus that was traditionally believed to consist uniformly of obligately aceticlastic methanogens.

The presence of ANME-1a-b in the SMTZ and in underlying sediment that is net methanogenic can be explained with different scenarios. The first one is that ANME-1a-b are indeed facultatively methanogenic, as proposed previously based on strong heterogeneity in *δ*^13^C of ANME-1-biomass in seep sediments (House et al., 2009), detection of ANME-1a-b *mcr*A transcripts in methanogenic sediment (Lloyd et al., 2011), and combined methanogenesis rate measurements and *mcr*A analyses across anaerobic methane-oxidizing and methanogenic sediment (Beulig et al., 2019). This environmental evidence has been supported by recent genomic detections of hydrogenase genes, that are potentially involved in hydrogenotrophic methanogenesis, across multiple ANME-1a-b and ANME-1c taxa (Laso-Pérez et al., 2023). Alternatively, AOM by ANME-1a-b may continue as a cryptic process in the presence of methanogenesis throughout the uppermost part of the methanogenesis zone. Our thermodynamic calculations indicate that a reversal of methanogenic CO_2_ reduction with H_2_ is thermodynamically favorable throughout bulk sediments of ODP Site 1230 (Figure 6), though the electron acceptor is unclear. As discussed earlier, the increase in dissolved barium indicates barite (BaSO_4_) dissolution in this part of the sediment column as a potential source of sulfate, whereas the very low Fe^2+^ and Mn^2+^ concentrations argue against a significant role of AOM coupled to metal reduction. Under this scenario, the organisms that were responsible for the production of the measured methane are unknown. While neither possibility can be ruled out, the available evidence from this and past studies supports ANME-1a-b contributing to the production of methane by CO_2_ reduction in the upper methanogenesis zone of ODP 1230.

The metabolism of ANME-1d, which replaces ANME-1a-b in deeper sediment layers of the methanogenesis zone, is even less understood than that of ANME-1a-b. This group, which was previously also referred to as “ANME-1-related group” and represents a poorly studied, sister family, or even sister order, of ANME-1a-b (Lever and Teske, 2015), has been found across a range of anoxic environments. These include hydrothermal vents in ultramafic settings (Kelley et al., 2005), deeply buried terrestrial coalbeds (Fry et al., 2009), marine gas hydrate sediments (Kormas et al., 2005), and tidal creek sediments (Edmonds et al., 2008). Given the sole detection of ANME-1d DNA and mRNA deep in the methanogenesis zone, a methanogenic lifestyle seems likely. This group could reduce CO_2_ to methane using electrons from IET and thus contribute to the observed strong *δ*^13^C-depletion of methane relative to DIC.

The predominant detection of *Methanothrix-mcr*A sequences below 20 mbsf, despite *δ*^13^C-DIC compositions that indicate mainly methanogenesis by CO_2_ reduction, and Gibbs energies of aceticlastic methanogenesis near thermodynamic equilibrium, is perplexing, given that members of *Methanothrix* are traditionally considered to be obligate aceticlasts. One explanation is that these sequences belong to inactive or dead cells. Yet, this explanation does not match the detection of rRNA of *Methanothrix* (Table 2), and is at odds with research suggesting that the vast majority of DNA from dead microorganisms is degraded over time scales of centuries in subsurface sediments (Torti et al., 2018). Instead, the *Methanothrix mcr*A and 16S rRNA sequences may not belong to (obligate) aceticlasts. While genomic data of *Methanothrix thermophila* indicate potential for hydrogenotrophic metabolism in this group (Smith and Ingram-Smith, 2007), methanogenesis involving H_2_ has never been shown for *Methanothricaceae*. Yet, more recent experiments with pure cultures have demonstrated that members of *Methanothrix* - including *Methanothrix harundinacea*, which *mcr*A sequences from ODP Site 1230 cluster with (Figure 5) - are capable of methanogenic growth by CO_2_ reduction using electrons received directly or through mineral intermediates from syntrophic partner organisms (Rotaru et al., 2014; Yang et al., 2019; Gao and Lu, 2021). Experiments involving rice paddy soils and lake sediments have provided additional evidence for CO_2_ reduction by *Methanothrix* in the environment (Holmes et al., 2017; Rotaru et al., 2019). Consequently, the observed *δ*^13^C-DIC and *δ*^13^C-methane compositions and dominance of *mcr*A sequences of *Methanothrix* may not be a contradiction, but instead match revised knowledge on the metabolic capabilities of *Methanothrix*.

## Conclusion

We provide the first complete community profile of active methane-cycling archaea in deep subseafloor sediments, and show based on DNA and RNA sequence data that anaerobic methane-cycling archaea are present throughout the SMTZ and methanogenesis zone of ODP Site 1230 in the Peru Trench. Of essential importance for the detection of *mcr*A, *mcr*A-mRNA, and 16S rRNA of methane-cycling archaea was the use of redesigned general *mcrA* primers and development of new group-specific *mcrA* and 16S rRNA gene primers. While these primers improved the detection sensitivity of methane-cycling archaea, they confirm the notion that methane-cycling archaea only account for a small fraction of deep subsurface microbial communities, even in AOM and methanogenesis zones (Lever, 2013).

Even though DNA- and RNA-based detections of methane-cycling archaea generally match the distributions of AOM and methanogenesis based on geochemical data, the detected phylogenetic groups appear at odds with the inferred dominant methane-cycling pathways. ANME-1, which are historically considered to be anaerobic methanotrophs, were detected to sediment depths that were > 10 m (ANME-1a-b) and > 100 m (ANME-1d) below the SMTZ. Based on published sedimentation rates for Site 1230 (0.25 mm yr.^−1^; Shipboard Scientific Party, 1988), these distances suggest the continued existence of ANME-1a-b and ANME-1d populations in methanogenic sediments for >40,000 and > 400,000 years after their burial below the SMTZ, respectively. Given the measured methane concentration and DIC-isotopic data, and that no other methane-cycling archaea were detected, a switch to methanogenesis by CO_2_ reduction offers the most parsimonious explanation for the occurrence of ANME-1a-b far below the SMTZ. Similarly, methanogenesis by CO_2_ reduction may sustain populations of ANME-1d in deeper layers, and also explain why members of *Methanothrix* – that were historically assumed to be aceticlastic - are pervasive throughout sediments that appear to be dominated by methanogenic CO_2_ reduction. Herein, the pathway of CO_2_ reduction remains unclear, but could bypass H_2_ as an electron source through direct electron transfer.

## Data availability statement

The original contributions presented in the study are publicly available. This data can be found here: All isotopic data (d13C-CH4, d13C-DIC) are included in Supplementary Table 1. All geochemical concentration data, including pH, are publicly available in D’Hondt et al. (2003). All nucleotide sequences can be retrieved from GenBank (mcrA: OQ603043-OQ603056; 16S rRNA: OQ658172-OQ658186).

## Author contributions

ML, MA, and AT designed the research. AT and K-UH obtained the samples. ML and K-UH produced the data. ML analyzed the data with input from MA and AT and wrote the manuscript with input from all co-authors. All authors contributed to the article and approved the submitted version.

## Funding

Sequencing was supported by the NASA Astrobiology Institute “From Early Biospheric Metabolisms to the Evolution of complex systems” and performed at the Josephine Bay Paul Center for Comparative Molecular Biology and Evolution at the Marine Biological Laboratory, Woods Hole, MA. ML was supported by a Schlanger Ocean Drilling Fellowship, and a University of North Carolina Dissertation Completion Fellowship.

## Conflict of interest

The authors declare that the research was conducted in the absence of any commercial or financial relationships that could be construed as a potential conflict of interest.

## Publisher’s note

All claims expressed in this article are solely those of the authors and do not necessarily represent those of their affiliated organizations, or those of the publisher, the editors and the reviewers. Any product that may be evaluated in this article, or claim that may be made by its manufacturer, is not guaranteed or endorsed by the publisher.

## Footnotes

^1^www.ncbi.nlm.nih.gov/blast

^2^http://www.arb-home.de/

^3^https://drive.google.com/drive/u/0/folders/1G8GeJuYsIX4MLv5-LaUQHD9f9F9rfIAu
