# Supplementary material for: Zonation of the active methane-cycling community in deep subsurface sediments of the Peru trench
Source: Front Microbiol. 2023 May 12;14:1192029. doi: 10.3389/fmicb.2023.1192029 (PMC10213550; doi:10.3389/fmicb.2023.1192029)
Supplement: Supplementary file 1 [file Data_Sheet_1.PDF]

SUPPLEMENTARY TEXT

Field Site. The Peru Trench is part of the larger Atacama Trench that is located between the continental South American Plate and the accretionary wedge of the oceanic Nazca Plate. It stretches parallel to the west coast of South America for 5,900 km, and is unique for a deep sea trench in that it combines slope deposits of mostly diatomaceous mud mixed with sediments from an accretionary wedge, high sedimentation rates, high organic carbon content in the sediment (Suess et al., 1988), and exceptionally high microbial activity in the sediment column (D'Hondt et al., 2004).

Sampling

The upper ~210 m were sampled with an Advanced Piston Corer (APC; D'Hondt et al., 2003): a drill is "advanced" to directly above the depth of interest, and then a 9.5-m piston core hydraulically thrust into underlying sediments (Graber et al., 2002). Below 210 m, the sediment was too firm for piston coring and was obtained by drilling with an Extended Core Barrel (XCB; Graber et al., 2002). APC sampling is preferable for microbiological analyses due to on average lower contamination of core interiors with drilling fluid; the interior of XCB samples from ODP Site 1230 showed remarkably low levels of contamination, however, and were suitable for microbiological studies (House et al., 2003). Three boreholes were drilled within close proximity to another (~20 m; D'Hondt et al., 2003).

Porewater samples for geochemical analyses were obtained from 20-40 cm long whole-round intervals using a hydraulic press as described previously (D'Hondt et al., 2003). For carbon isotope analyses, 5-mL subsamples were frozen in precombusted glass vials.

For molecular biological analyses, 5-cm whole-round intervals of cores were frozen at -80°C. Only sediment from the nearly contamination-free interior was used (House et al. 2003, Lever et al. 2006).

Porewater geochemical gradients

We used previously published depth profiles of porewater alkalinity, sulfate, CH_4, H_2, formate and acetate concentrations (Fig. 2A-E; D'Hondt et al., 2003). Due to outgassing during core retrieval, measured CH_4 concentrations below 15-20 mbsf were underestimates of *in situ* concentrations. Methane concentrations below the saturation depth (~28 mbsf) were hence calculated using the model for CH_4-seawater-porous media at H-L-equilibrium (Sun and Duan, 2007) at *in situ* temperature, pressure, and salinity, and assuming a uniform pore size of 1.0 μm. Since the assumed pore size of 1.0 μm most likely overestimates *in situ* pore size in the highly compacted clay sediments, our calculations probably underestimate *in situ* CH_4 concentrations.

Supplementary Table 1. Overview of measured (A) $\delta^{13}C-CH_4$ (based on $\delta^{13}C-C_1$) and (b) $\delta^{13}C$-DIC values and their differences. All $\delta^{13}C$-DIC data are from Meister et al. (2007). Note: we omit $\delta^{13}C-CH_4$ values from below 12 mbsf (gray font) in Borehole B from further analyses due to poor data quality that includes three outliers (13.95 mbsf, 21.45 mbsf, 22.16 mbsf).

(A)

Borehole	Depth (mbsf)	$\delta^{13}C-C_1$ (in ‰)[a]
B	1.2	-64.21
B	3.0	-65.09
B	4.5	-65.37
B	6.0	-64.68
B	7.5	-68.32
B	9.0	-75.33
B	12.0	-74.53
B	13.95	-57.28
B	15.45	-76.00
B	16.85	-71.04
B	18.45	-75.186
B	21.45	-63.91
B	22.16	-59.56
B	28.45	-72.79
A	29.75	-70.48
A	36.3	-70.57
A	40.8	-72.90
A	58.6	-70.02
A	62.1	-69.57
A	68.2	-67.8
A	71.8	-66.28
A	77.8	-67.70
A	81.1	-68.21
A	87.1	-67.3
A	93.8	-66.04
A	95.3	-65.03
A	101.75	-66.26
A	104.8	-66.62
A	112.87	-65.42
A	115.67	-59.44
A	123.99	-65.1
A	126.84	-63.90
A	130.6	-65.6
A	141.6	-65.9
A	161.78	-65.66
A	169.0	-66.16
A	188.6	-66.37
A	248.01	-64.67

(B)

Borehole	Depth (mbsf)	δ^{13}C-DIC (in ‰)[b]
A	1.35	-10.4
A	2.85	-11.9
A	4.44	-12.0
A	6.15	-12.6
A	7.65	-13.2
A	9.15	-8.4
A	12.15	-3.4
A	13.65	-0.9
A	15.65	2.4
A	17.15	4.0
A	18.65	5.6
A	21.65	7.0
A	23.15	7.8
A	28.15	9.5
A	31.15	10.4
A	36.15	13.1
A	40.65	13.1
A	58.65	14.6
A	62.15	15.3
A	68.25	15.9
A	71.65	16.0
A	77.65	16.3
A	81.15	16.6
A	87.15	17.1
A	92.15	17.1
A	96.45	17.2
A	100.15	17.7
A	106.15	17.5
A	109.65	17.3
A	115.72	17.8
A	123.47	19.6
A	126.89	18.4
A	130.65	18.4
A	140.1	17.5
A	161.63	17.3
A	169.05	18.2
A	188.65	16.3
A	246.36	15.1

Supplementary Table 2. Overview of standard Gibbs energies ($\Delta G_f°$), standard enthalpies ($\Delta H_f°$), and standard molal volumes ($\Delta V_f°$) of formation that were used to calculate in situ Gibbs energies of methanogenesis reactions from H_2/CO_2, acetate, and formate.

**Compound**	**$\Delta G_f°$ (kJ mol^{-1})**	**$\Delta H_f°$ (kJ mol^{-1})**	**$\Delta V_f°$ (cm^3 mol^{-1})**	**Reference**
proton (H^+)	0.0	0.0	0.0	Shock et al. (1997)
hydrogen (H_2)	17.57	-4.16	25.2	Wagman et al. (1982), Shock and Helgeson (1990)
water	-237.18	-285.83	18.0	Amend and Shock (2001)
bicarbonate	-586.9	-692.0	24.6	Wagman et al. (1982), Shock et al. (1997)
acetate	-369.41	-486.42	40.5	Shock and Helgeson (1990)
formate	-351.04	-425.71	26.16	Shock and Helgeson (1990)
methane	-34.47	-87.96	37.30	Shock and Helgeson (1990)

Supplementary Figure 1. Sulfate and methane porewater concentration profiles in Borehole A. Shaded area indicates approximate depth interval of the SMTZ, where most AOM takes place, in this borehole (7 to 9 mbsf). Dashed lines were hand-drawn in for easier visualization.

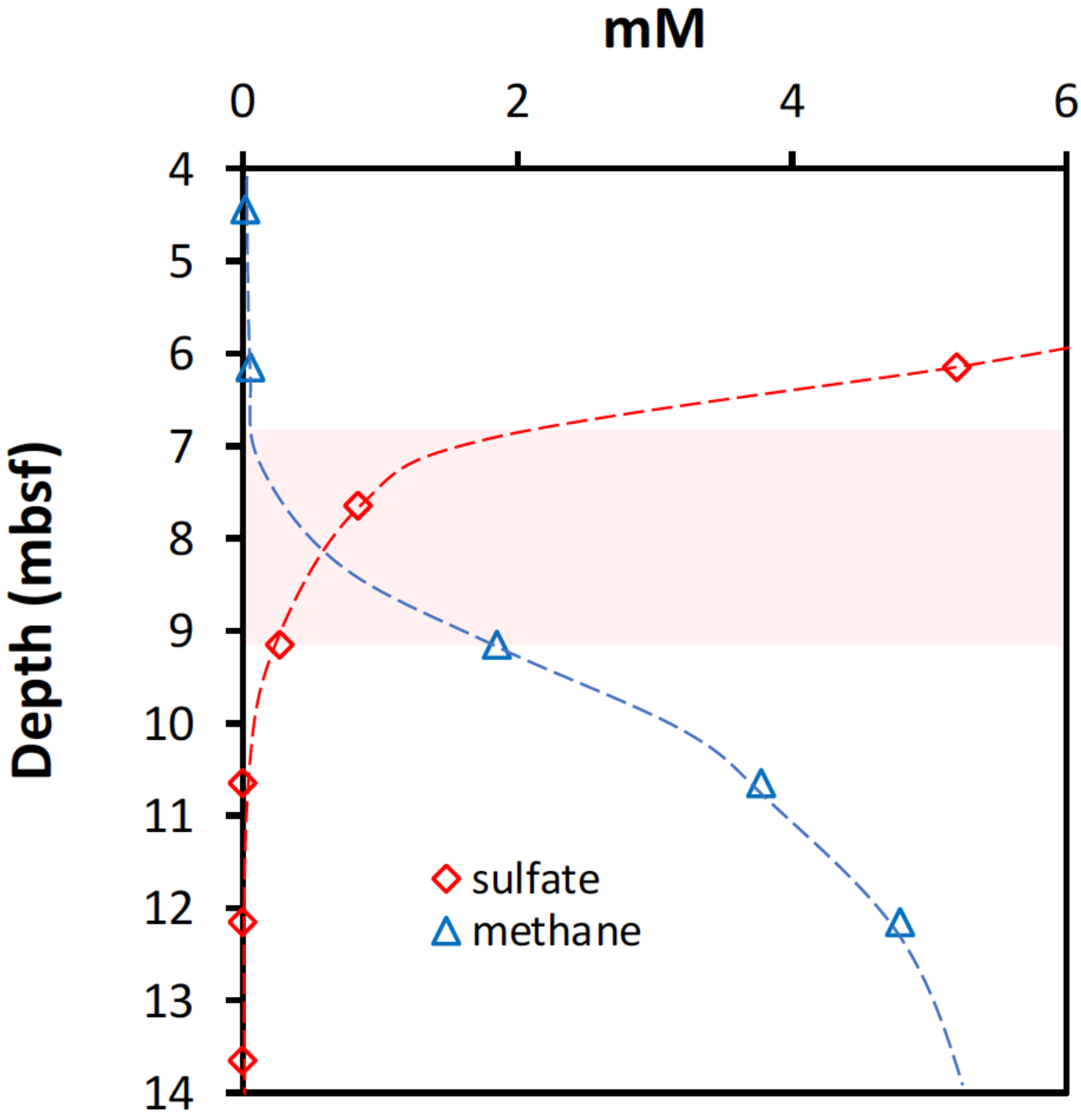

Supplementary Figure 2. Porewater formate and acetate concentrations in the upper 20 mbsf at ODP Site 1230. All data are from borehole A.

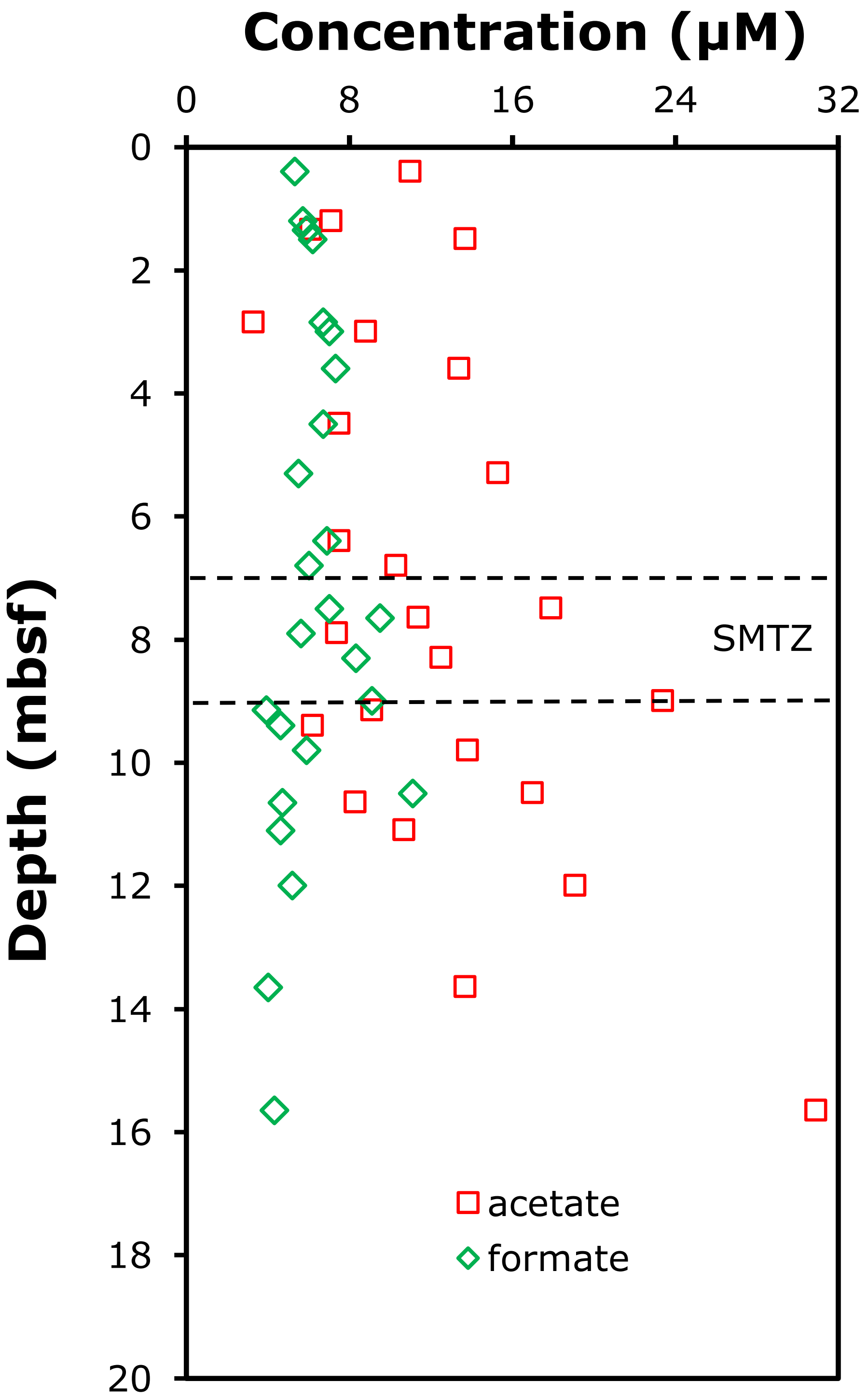

Supplementary Figure 3. Bootstrap phylogenetic tree based on nucleotide sequences of 16S rRNA. Created using Jukes-Cantor correction in ARB neighbor-joining. All sequences from this study are in magenta. Sequences recovered with new group-specific 16S primers all fall within the *Methanomicrobia*. All other sequences were obtained with the general archaeal ARC 8F-915R primer combination. For orientation, we include one sequence of ANME-1 (Methanophagales; in blue), a group whose 16S sequences could not be detected, despite the use of group-specific 16S primers.

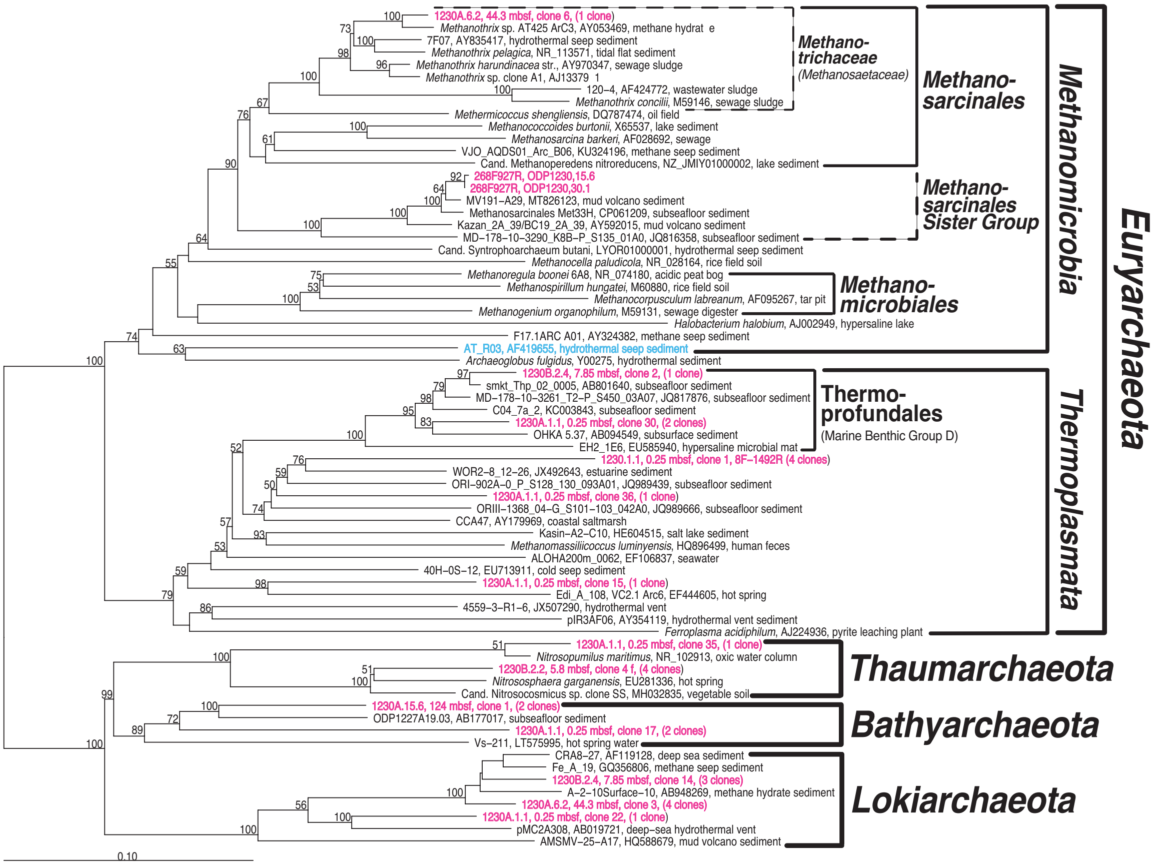

SUPPLEMENTARY REFERENCES

Amend, J. P., Shock, E. L. 2001. Energetics of overall metabolic reactions of thermophilic and hyperthermophilic archaea and bacteria. *FEMS Microbiol. Rev.* 25, 175-243.

D'Hondt, S. L., Jørgensen, B. B., Miller, D. J., and Shipboard Scientific Party. (2003). 1. Leg 201 Summary. Proc Ocean Drilling Program, Init. Repts 201.

D'Hondt, S., Jørgensen, B. B., Miller. D. J., Batzke, A., Blake, R., Cragg, B. A., et al., (2004). Distributions of microbial activities in deep subseafloor sediments. Science 306, 2216-2221.

Graber KK, Pollard E, Jonasson B, Schulte F (2002) Overview of Ocean Drilling Program engineering tools and hardware. *ODP Tech Note 31*.

House, C. H., Cragg, B. A., Teske, A., and the Leg 201 Scientific Party. (2003). Drilling contamination tests during ODP Leg 201 using chemical and particulate tracers. In: ODP Leg 201, Scient. Results.

Meister, P., McKenzie, J. A., Vasconcelos, C., Bernasconi, S., Frank, M., Gutjahr, M., and Schrag, D. P. (2007). Dolomite formation in the dynamic deep biosphere: results from the Peru Margin. Sedimentology 54, 1007-1031.

Shock, E. L., and Helgeson, H. C. (1990). Calculation of the thermodynamic and transport properties of aqueous species at high pressures and temperatures: standard partial molal properties of organic species. *Geochim. Cosmochim. Acta* 54, 915-945.

Shock, E. L., Sassani DC, and Willis M, Sverjensky DA. (1997). Inorganic species in geologic fluids: correlations among standard molal thermodynamic properties of aqueous ions and hydroxide complexes. *Geochim. Cosmochim. Acta* 61, 907-950.

Suess, E., von Huene, R., et al., 1988. Proc. ODP, Init. Repts., 112: College Station, TX (Ocean Drilling Program).

Sun, R., and Duan, Z., (2007). An accurate model to predict the thermodynamic stability of methane hydrate and methane solubility in marine environments. Chem. Geol. 244, 248-262.

Wagman, D. D., Evans, W. H., Parker, V. B., Schumm, R. H., Halow, I., Bailey, S. M., Churney, K. L., and Nuttall, R. L. (1982). The NBS tables of chemical thermodynamic properties: Selected values for inorganic and C1 and C2 organic substances in SI units: *J. Phys. Chem. Ref. Data*, 11, 392 p.
